# Prescription Department

**Published:** 1893-01

**Authors:** 


**Hoarseness and Catarrhal Coughs.—**

R. Ammonium acetate, 3 parts.
Potassium bromide, 3 "
Tincture of belladonna, 1 1-2 "
Tincture of aconite, 5 "
Infusion of balsam of tolu, 150 "
Syrup of baisam of tolu, 50 "

Sig. A tablespoonful is to be taken every three or four hours.—*"L'osservatore."*

**Radical Cure of Hæmorrhoids.—**

R. Ext. fl. ergot.

Acid caıbolic pura, aa dr. ss.

Sig. Inject only non-indurated, and situated well above the sphincter muscles.—*J. W. Charles, M. D.*

**For Convulsions in Teething Children.—**

R. Potass. bromid.
Amm. bromid, aa scruple ij.
Fl. ext. gelsem.
Fl. ext. valerian, aa gtt. xx
Aquae et syr. simplex, aa oz. ss.

M. Sig. Teaspoonful every one-half to one or two hours.—*John J. Gage, M. D.*

**For Dysentry.**

R. Elixir vitirol, oz. j.
Elixir paregoric, oz. ij.
Aqua camphor, oz. v.
Olei menthæ piperiti, gtt. xxv.

M. Sig. Tablespoonful through a quill every three hours in a wineglass of water. Wash the mouth well with bicarb-soda after each dose.—*Dr. Wm. C. Hewell.*

**Nervous Cough.**

R. Fl. ext. hyoscyamus, dr. iv.
Tr. gelsemium, dr. iij.
Potassa bromide, grs. lx.
Aqua pera, oz. iv.

M. Sig. Half teaspoonful three times a day.—*J. C. Fear, M. D.*

**Nervous Headache.**

R. Chloral hypratis, dr. iiss.
Potassi bromidi, dr. iss.
Tinct. valeriani.
Syrup simplicis, aa oz. j.

M. Sig. One teaspoonful in water every two or three hours until relieved.—*L. W. Evans, M. D.*

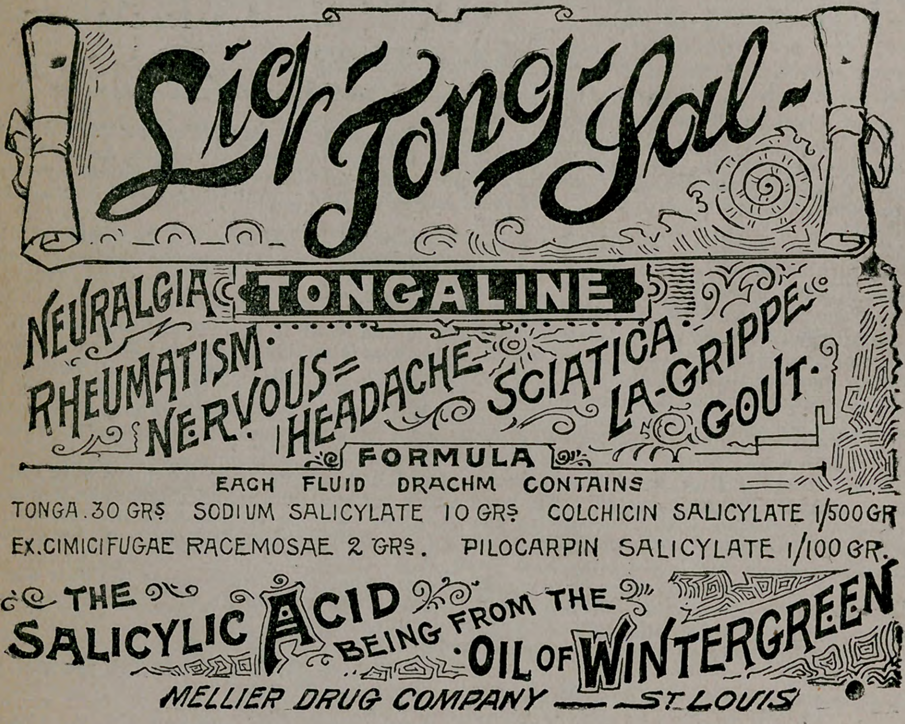

FOR DYSMENORRHŒA.

R. Liq. potassæ acetatis, oz. iss.
Spts. chloroformi, dr. iv.
Spts. ammon. arom., dr. iij.
Elx. simplex.

M. Sig. Teaspoonful in half cup hot water three to six times daily during painful menstruation.—*C. P. Donelson, M. D.*

GONORRHŒA.

R. Ol sandal wood, dr. ij.
Liq. potassæ, dr. ij.
Sacch. alb., dr. iij.
Gum acacia, dr. iij.
Aqua cinnamon, oz. vj.

M. ft. sol. Sig. Teaspoonful ter in die.—*Dr. R. C. McCann.*

PHARYNGITIS.

R. Potassæ chloratis pulveris, dr j.
Acidi hydrochlorici U. S. P., oz. jss.
Misce et adde.
Tincture ferri chloridi, dr. ij.
Aquae q. s. ad., oz. iv.

Sig. Teaspoonful every two hours, with no water.—*Prof. Waugh.*

SPECIFIC FOR AGUE.

R. Quinine sul., dr. iss.

Sulphate ferri, dr. i.

Oil sassafras, } aa gtt. xxx

Oil blk pepper, }

Arsenious acid, gr. ij.

Mix et fiat pill, No. xxx.

Sig. One pill three times a day at meal time.—*C. H. B. Gile, M. D.*

FLATULENT DYSPEPSIA.

R. Bismuth salicylat, part 2.
Magnesiæ calcin, part 2.
Pulv. carbonis salicis, part 3.
Ol. anisi. part 1.

M. Sig. Dose: One teaspoonful half an hour or an hour before each meal.—*Dr. Huchard.*

FOR SCARLET FEVER.

R. Acid salicylic dr. ij.
Tr. aconiti rad., gtt. xij.
Inf. digitalis, oz. iss.
Spt. ammon. aromat., oz. ss.
Syr. aurant. cort. oz. iij.
Aquae distil., oz. j.

M. Sig. Teaspoonful for a child five years old, every three hours.—*Dr. Bedford Brown.*

